# Physical activity, sleep, dietary habits, and screen time in Bogotá’s children post-pandemic lockdown: a mixed-methods study

**DOI:** 10.1080/17482631.2026.2683936

**Published:** 2026-06-13

**Authors:** Shirley Castrellon-Vergara, Blanca Nathalia Rueda-Alfonso, María Alejandra Palacios-Ariza, Fabian Ricardo Guevara-Santamaría

**Affiliations:** a Programa de Especialización en Pediatría - Facultad de Medicina, Fundación Universitaria Sanitas, Bogotá D.C., Colombia; b Unidad de Investigación, Fundación Universitaria Sanitas, Bogotá D.C., Colombia; c Versania Primera Infancia, Grupo Keralty, Bogotá, D.C., Colombia

**Keywords:** Child health, lifestyle, COVID-19, dietary habits, physical activity, screen time, sleep

## Abstract

**Purpose:**

This study explored how Bogotá’s children reconfigured their post-lockdown routines as part of their lived experience after returning to in-person activities.

**Methods:**

A mixed-methods design was applied to capture behavioral patterns and their meanings. Quantitatively, 217 children aged 8–12 years attending pediatric clinics (Oct 2022–Sep 2023) completed validated questionnaires assessing physical activity, sleep, diet, and screen exposure. Qualitatively, semi-structured interviews were conducted with 15 children selected through purposive sampling to deepen insight into their daily routines and perceptions. Quantitative data were analyzed descriptively and by sex, while thematic analysis of interviews provided contextual interpretation. Integration occurred through a narrative weaving approach.

**Results:**

Quantitative findings indicated moderate physical activity (66% met ≥ 3 days/week), widespread sleep disturbances (49.7%), recreational screen use (median= 6 hours/day), and poor dietary intake (>90% consumed fewer than two vegetable servings daily). Narratives revealed that play was often replaced by structured adult-imposed activities, screen time served as both emotional refuge and behavioral reward, and sleep was self-managed through distractions such as eating or watching TV. Children viewed these habits as normal despite feelings of fatigue, frustration, and a longing for autonomy.

**Conclusion:**

Children's post-lockdown routines reflect adaptation rather than behavioral lapses, requiring interventions focus on emotional regulation, time balance, quality rest, and active play (not merely a return to pre-pandemic norms).

## 1. Introduction

The COVID-19 pandemic led to drastic changes in children’s daily lives, largely due to prolonged school closures and mobility restrictions (Acevedo-Rincón & Flórez Pabón, 2022; Göl-Güven et al., 2025; Liu et al., 2021; Silva et al., 2020). In Colombia, lockdown measures lasted over five months, and limitations on outdoor activity for children remained in place until mid-2022 (Prada et al., 2022). Although these interventions reduced viral spread, they also disrupted children's routines, raising concerns about the long-term consequences for their physical and mental health (Al-Mulla & Mahfoud, 2022; Graber et al., 2021). Evidence from multiple countries indicates a marked decline in physical activity during lockdown, accompanied by increases in screen use, sedentary behaviour, and altered sleep and eating patterns (Genin et al., 2021; López-Bueno et al., 2020). Unstructured play often replaced organised sports but did not offset overall activity loss (Nathan et al., 2021). Screen exposure, both for learning and entertainment, expanded significantly, often exceeding recommended limits and interfering with sleep and social interaction (Łuszczki et al., 2021; Narang et al., 2024; Shenoy et al., 2021).

Nutrition-related behaviours also shifted, with reports of irregular meal patterns and increased consumption of snacks and sugary drinks (Bartha et al., 2022; Teixeira et al., 2021). Several studies reported a decrease in regularly-scheduled meals, and up to 42% of surveyed parent in a Canadian study reported increased eating in their children (Carroll et al., 2020; Pfefferbaum et al., 2024). Sleep routines were affected by irregular schedules, delayed bedtimes, and bedtime screen use, factors linked to reduced sleep quality and poor daytime functioning (Bartha et al., 2022; Green et al., 2017). Studies from diverse contexts, including India, Brazil, Hungary, and Australia, have documented psychosocial effects such as emotional distress, social withdrawal, and behavioural changes, particularly in low-income families (Amaral de Andrade Leão et al., 2025; Bartha et al., 2022; Munasinghe et al., 2020; Rajesh et al., 2021). These effects were often associated with increased digital engagement and diminished peer interaction during confinement, contributing to prolonged screen exposure and reduced opportunities for physical and social activity (Pedrouzo & Krynski, 2023).

Despite extensive documentation of lifestyle disruptions during the pandemic, less is known about how children’s behaviours have evolved since the return to in-person schooling and the easing of restrictions—especially in Latin American urban settings like Bogotá. Most post-pandemic research focuses on short-term outcomes or parental reports, with limited exploration of children's own perspectives on their daily routines and lifestyle choices. To address this gap, this study aimed to describe the current lifestyle habits of children aged 8 to 12 in Bogotá, focusing on physical activity, sleep, screen exposure, and eating habits following the relaxation of COVID-19 containment measures. Using a mixed-methods approach, the study integrated data from standardised questionnaires with children's own narratives.

## 2. Methods

A mixed explanatory sequential design with a pragmatic approach was adopted, recognising that schoolchildren’s lifestyle behaviours following the COVID-19 lockdown required both quantitative objectivity and qualitative depth to be explored adequately (Creswell & Plano Clark, 2018; Fetters et al., 2013). The design, collection, and analysis of data were based on validated quantitative tools and a qualitative component using semi-structured interviews. Initially, all participants completed the questionnaires in outpatient consultations, where aspects of physical activity, sleep, diet, and screen exposure were assessed. Afterward, a subset of participants was selected through purposive and convenience sampling. Integration was achieved at three levels. At the design level, the sequential structure allowed the qualitative phase to be informed by preliminary quantitative findings (building). At the methods level, integration occurred through connecting (selecting interview participants based on questionnaire responses) and merging (comparing and interpreting findings from both datasets). Finally, at the interpretation level, a weaving narrative approach was used to integrate qualitative insights with quantitative trends, enhancing explanatory power.

In Colombia, nationwide pandemic restrictions began with a mandatory stay-at-home order that took effect on 25 March 2020, limiting outings to essential purposes such as buying food and medicines, caring for dependents/pets, and accessing healthcare. Schools (public and private) suspended in-person classes and shifted to remote modalities starting in March 2020, with reopening occurring gradually during 2021 as in-person attendance resumed under phased return strategies. While mobility restrictions were eased over time (including a transition away from strict nationwide lockdowns later in 2020), mask requirements remained in place in public settings for a prolonged period. Data collection took place in the outpatient clinic of three paediatric hospitals in Bogotá, Colombia, between October 2022 and September 2023.

### 2.1. Participants

Eligible participants were children aged 8 to 12 years attending outpatient well-child visits. Children with chronic medical conditions (e.g. asthma, diabetes), psychiatric disorders (e.g., depression, attention deficit hyperactivity disorder, autism spectrum disorder), or significant physical, sensory, or cognitive impairments (e.g. cerebral palsy, visual/hearing disabilities, trisomy 21) were excluded if such conditions limited their ability to complete study assessments. This study was approved by the Research Ethics Committee of the Fundación Universitaria Sanitas (CEIFUS 3095-22). Informed consent was obtained from guardians and assent from participating children prior to enrolment.

### 2.2. Quantitative lifestyle assessment

Sociodemographic data included age, sex, school type (public/private), socioeconomic stratum (SES), and parental education. Health-related and lifestyle outcomes were assessed using standardised instruments. Physical activity was measured with the Physical Activity Questionnaire for Children (PAQ-C), designed for self-report in children aged 8–14 years (Kowalski et al., 2004). It provides a composite score from nine items covering activity during PE classes, recess, after school, and weekends, plus an item on illness or events limiting activity. The Spanish version used has been validated for Colombian children, showing acceptable consistency and reliability (Herazo-Beltrán & Domínguez-Anaya, 2012). The total score is calculated as the average of the items and ranges from 1-5, with higher scores indicating higher levels of habitual physical activity.

Sleep behaviours were assessed via the BEARS sleep screening tool, a brief clinical questionnaire covering five domains: Bedtime problems, Excessive daytime sleepiness, Awakenings, Regularity/duration, and Snoring (Bruni et al., 1996). It was administered in parent–child interviews to screen for sleep disturbances. Additionally, dietary behaviours were assessed using items adapted from the updated technical document of the Colombian Food-Based Dietary Guidelines for populations over 2 years of age (Instituto Colombiano de Bienestar Familiar (ICBF) & Organización de las Naciones Unidas para la Alimentación y la Agricultura FAO, 2015). These included the number of daily meals, frequency of breakfast consumption, intake of fruits, vegetables, sugary beverages, and nighttime snacks. Screen time exposure was evaluated by differentiating hours spent using screens for academic versus recreational purposes, based on international paediatric guidelines (Hill et al., 2016; Reid Chassiakos et al., 2016). All questionnaires were administered during face-to-face visits and completed with the support of trained paediatric research staff. The three questionnaires have either been developed by the Colombian government or have been endorsed by the Ministry of Health in official documents.

### 2.3. Qualitative semi-structured interviews

After completing the quantitative questionnaires, a purposive subsample of participants was selected for semi-structured interviews to expand and contextualise initial findings. Selection was based on participants’ willingness and the clarity and richness of their verbal responses during questionnaire administration. The strategy prioritised children with communicative fluency and the ability to express reflective insights about their routines, behaviours, and post-lockdown experiences, ensuring perspectives relevant to the research questions.

The interview guide explored daily life from the child's perspective, focusing on activities and routines after the resumption of in-person schooling and social life. Questions addressed engagement with physical activity, sleep, eating habits, screen use, and time organisation (Supplemental 1). Children were also asked to describe meaningful or enjoyable aspects of their daily lives and reflect on personal practices and preferences. The guide allowed for open-ended responses while maintaining thematic consistency.

Interviews were conducted in outpatient settings with a parent or guardian present, lasted 20–30 minutes, and were audio-recorded with assent. Transcriptions were completed verbatim using Amberscript and manually reviewed by the research team. All interviews followed a standardised protocol and were carried out by trained paediatric researchers.

### 2.4. Statistical methods

Categorical variables were described using relative and absolute frequencies. Normality was determined using the Shapiro-Wilk test and given the non-normality of the study variables, all were presented as medians and interquartile ranges. We carried out exploratory analyses on differences in screen times, physical activity, sleep disturbances and nutritional features by sex. We also analysed differences in screen times based on whether the child had access to a cellphone. All hypothesis testing used a threshold of 0.05 for significance. Analyses were carried out using R (v.4.2.2).

### 2.5. Qualitative analysis

Interview transcripts were analysed using deductive-inductive thematic analysis, following Braun and Clarke’s six-phase approach (Braun & Clarke, 2022). This method was selected for its theoretical flexibility and compatibility with our pragmatic mixed-methods design, allowing for both deductive (theory-driven) and inductive (data-driven) coding orientations to complement the quantitative findings.

The analysis process involved: (1) familiarisation through reading and re-reading transcripts; (2) systematic data coding using a dual approach. Initially, deductive coding was applied based on the interview guide's thematic domains (daily routines, physical activity, rest and sleep, screen use, eating habits, and children's subjective experiences), allowing direct alignment with quantitative categories for triangulation. Subsequently, all transcripts were re-coded inductively to capture emerging patterns and lived experiences not anticipated in the initial framework; (3) generating themes from the complete coded dataset through systematic comparison across participants, from which two additional categories emerged: Time Management and Interpersonal Relationships; (4) reviewing and refining all themes against coded extracts and the full dataset; (5) defining and naming final themes to capture their central organising concepts; (6) producing the analytic narrative. The lead researcher maintained a reflexive journal throughout to document analytic decisions and interpretations.

Manual coding was conducted using ATLAS.ti v25, with analytical memos maintained to ensure reflexivity. Thematic saturation was considered reached after three consecutive interviews produced no new insights. The research team followed a dialogical approach, continuously reviewing and refining categories to ensure conceptual clarity and coherence with the study’s interpretive goals.

## 3. Results

**Figure 1.**
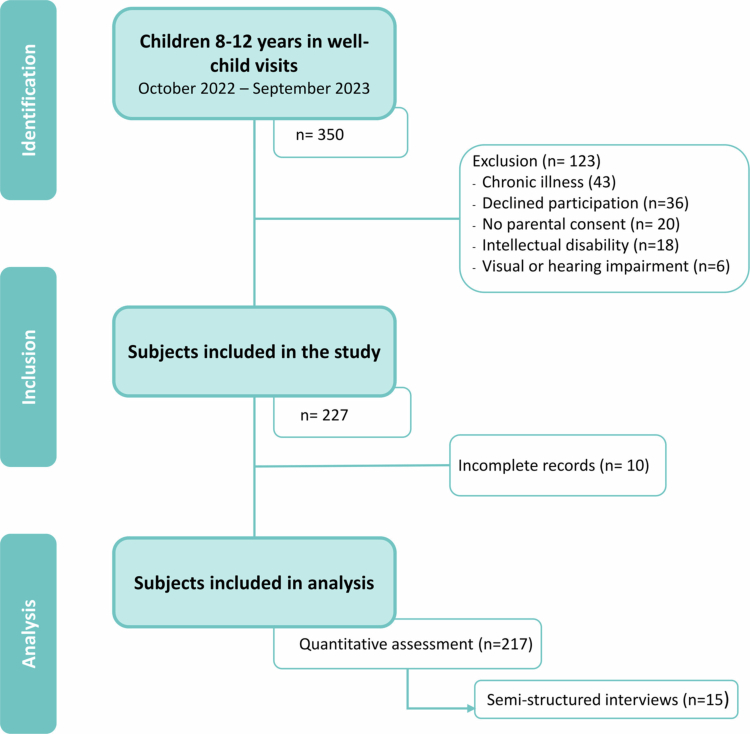
Patient flowchart: recruitment and subject selection process.

A total of 350 children were initially assessed for eligibility, 123 of whom were excluded (35.1%). Chronic illnesses, including type 1 diabetes mellitus, asthma, and epilepsy were the cause of exclusion in 43 children. Eighteen children were excluded due to intellectual disabilities associated with cerebral palsy (*n* = 3), genetic syndromes (*n* = 5), and autism spectrum disorders (*n* = 10). Visual or hearing impairment was the least frequent cause of exclusion with only 6 children. Despite child assent, 20 children were excluded due to a lack of parental consent. A further 36 children declined to participate. Excluded children were within the same age range (8-11 years) and had a similar sex distribution as that of the final sample (female sex 52.8% vs 50.7%, respectively) (Figure 1). Of the remaining 227 subjects, 10 were excluded due to incomplete data (neglected to answer some of the items in the questionnaires). This left 217 subjects which were ultimately included in the quantitative analysis. Semi-structured interviews were conducted in 15 of them.

The median age of the subjects was 10 years (IQR = 9–11), with females being slightly older than males (Mann-Whitney U *p*-value = 0.025). Females were significantly more likely to be enroled in secondary school, while males remained predominantly enroled in primary education (Chi-squared *p*-value = 0.033). Regarding socioeconomic status, over 95% of the participants belonged to the low-middle strata. Most children attended public schools and were engaged in in-person education programs (96%). No statistically significant sex-based differences were identified in nutritional status, biometric parameters (weight, height, BMI), or access to personal cellphones (Table I). The purposive subsample for semi-structured interviews consisted of 8 females and 7 males. These children had a median age of 11 years (IQR = 10–11) and demonstrated strong communication skills.

**Table I.** Sociodemographic and biometric characteristics of participating children aged 8–12 years, stratified by sex.

Variable	Total (*n* = 217)		Female (*n* = 110)		Male (*n* = 107)		*p* value
	n	%	n	%	n	%	
***Socioeconomic Stratum***							
Low	130	59.91	66	50.77	64	49.23	0.796*
Middle	77	35.48	40	51.95	37	48.05	
High	10	4.61	4	40	6	60	
***Current schooling***							
Primary (1st-5th grade)	155	71.43	71	45.81	84	54.19	**0.033**
Secondary (6th-11th grade)	62	28.57	39	62.9	23	37.1	
***Type of school***							
Public	128	58.99	63	49.22	65	50.78	0.702
Private	89	41.01	47	52.81	42	47.19	
***In-person education***	209	96.31	105	50.24	104	49.76	0.722*
***Nutritional status***							
Underweight	13	5.99	3	23.08	10	76.92	0.369
Risk of underweight	22	10.14	11	50	11	50	
Adequate BMI for age	135	62.21	71	52.59	64	47.41	
Overweight	34	15.67	18	52.94	16	47.06	
Obesity	13	5.99	7	53.85	6	46.15	
***Cellphone ownership***	90	41.47	48	53.33	42	46.67	0.069
	**Median**	**(Q1-Q3)**	**Median**	**(Q1-Q3)**	**Median**	**(Q1-Q3)**	***p* value**
***Age in years***	10	9-11	10	9-11.75	9	8-11	**0.025**
***Weight (kg)***	34	27-41	35	26.77-43.75	32.9	27.75-38.25	0.162
***Height (cm)***	140	132-148	140	132-150	140	132.5-144	0.303
***Body Mass Index (kg/m2)***	17.1	15.3-19.3	17.36	15.3-19.65	16.71	15.24-19.06	0.159

^*^ Fisher's exact test, all others Chi-squared test for categorical variables. *P* values for continuous variables correspond to Mann-Whitney U test. Q1-Q3: quartiles.

### 3.1. Physical activity

Among the total sample, 70.1% (*n* = 152) of children reported engaging in structured physical activities such as soccer, skating, swimming, or other organised sports during the week. Notably, 143 children (65.9%) met the recommended frequency of physical activity ≥3 times/week. The median PAQ-C score was 3 for both males and females, but the interquartile range was wider in males (IQR = 2–4) than in females (IQR = 2–3). Despite identical medians, this distribution difference was statistically significant (Mann-Whitney U *p* = 0.012), indicating higher activity scores were more frequent among males.

While quantitative data show moderate activity levels, children's accounts reveal tension between desire and ability to be active. Some had full weekly sport schedules arranged by caregivers, suggesting structured access. Others wanted more activity but were limited by time, homework, or lack of opportunity. For many, activity occurred mainly through spontaneous play at school or with siblings, rather than as a formal, sustained habit. These unstructured forms offered compensatory opportunities, especially when structured options were lacking. A few children also expressed interest in competitive sports but lacked the means or knowledge to begin, revealing a motivational gap not captured by the PAQ-C.

This variability suggests physical activity is less a stable habit than a reflection of family organisation, school dynamics, or socioeconomic conditions (Table II). Qualitative data also revealed emotional dimensions: some associated physical activity with joy and freedom, while others described frustration at not being able to participate as much as they wished. Thus, physical activity emerged not only as a health-related behaviour but also as an emotional experience, influenced by adult-managed routines and shaped by external responsibilities.

**Table II.** Joint display of lifestyle-related quantitative findings, qualitative themes, and representative child quotes.

Lifestyle Category	Key Quantitative Findings	Themes	Representative Quotes
Physical Activity (PAQ-C)	Median Physical Activity Score (Q1-Q3): 3 (1-2);	Unstructured play is the main form of physical activity.	*“Sometimes I run or play during recess, and when I’m not at school, I occasionally go to the park with my parents.* *”* (P8)
	Males (3; IQR = 2–4) showed higher PAQ-C scores compared to females (3; IQR = 2–3), indicating greater habitual physical activity (Mann Whitney U *p*-value = 0.012).	Some children follow intensive, structured sports routines.	*“I play soccer on Mondays and Thursdays, basketball on Wednesdays and Fridays, and tennis on Tuesdays and Saturdays.”* (P6)
		Others report low activity levels but wish to be more active.	*“I don't do much exercise, but I would like to start a competitive sport.”* (P12)
Sleep (BEARS)	49.7% of participants exhibited disturbances in at least one sleep domain (Figure 2).	Children follow more flexible sleep schedules on weekends.	*“My mom is making me go to bed at nine because I have to get up early for school, but I go to sleep at eleven at night on weekends.”* (P13)
	No statistically significant differences were found between males and females.	Many children use distractions like screens, reading, or eating to fall asleep.	*“I eat and read because I’m not sleepy, I distract myself with other things like the TV. And then that’s when I get sleepy.”* (P3)
		Difficulties in initiating sleep are frequently reported despite perceived rest.	*“I stay in bed for about an hour before falling asleep. But when I wake up the next morning, I feel very well rested.”* (P2)
Screen Exposure (AAP recommendation)	While the total number of screen time hours was similar between females and males (Median = 6), males had a slightly lower IQR (4–7) compared to females (5–8), and this difference was statistically significant (Mann-Whitney U *p*-value = 0.033).	There is a consistent pattern of increased screen time among children.	*“I watch almost eight hours of television a day.”* (P4)
	No significant sex differences were observed in screen time for educational purposes (*p* = 0.535).	Screens are used for learning and entertainment	*“During the week I’m learning to play the trombone through television, and on weekends I watch about six hours of movies.”* (P10)
	However, females reported significantly more hours of entertainment-related screen use (Median = 3; IQR = 2–4) compared to males (Median = 2; IQR = 1–3), with this difference being significant (*p* < 0.001).	Parents frequently use screen time as a reward for task completion.	*“When I finish all my schoolwork for the following week on Saturday, my mom lets me watch TV as a reward for my work.”* (P14)
Eating Behaviours (ICBF National Guidelines)	Most children reported a low intake of fruits and vegetables, with 67.7% consuming fewer than two fruit servings and 90.3% consuming fewer than two vegetable servings daily. A statistically significant difference by sex was found in vegetable intake: males were more likely to consume fewer than two servings of vegetables per day (52.0%) compared to females (48.0%) (Chi-squared *p* value = 0.026).	Perception–practice mismatch: belief in healthy eating vs. reported behaviours.	*“Lunch is always a surprise, I don’t know what I’m going to eat because it’s not predetermined. What I do know is that they give us something balanced: crispy chicken, ajiaco, bandeja paisa, among others.”* (P1)
	There were no significant differences by sex in the consumption of soft drinks, water, fruit, or nighttime snacks. The median number of daily meals was 5 for both sexes, with no statistical difference.	Sweets, processed foods, and bakery items are frequently included in daily consumption.	*“We eat well in the afternoons, if we’re hungry we tell my mom and she buys us a snack, for example: bread from the bakery, gelatin, rice pudding, or a dessert.”* (P14)
		Water is often replaced by sugar-sweetened beverages.	*“I only drink juice or soda when I’m thirsty. I don’t like drinking water, I feel like it has no taste.”* (P11)
Time Management (emergent)	(No quantitative data)	Children describe overscheduled daily routines with little free time. Many children report feeling tired.	*“Sometimes I’m falling asleep at 11 or 12 during the day because my routine starts really early and ends very late.”* (P7)
		Academic and extracurricular activities often extend into the evenings and weekends.	*“I have English and German classes almost every weekday in the evenings… On weekends I have volleyball and tennis lessons. There’s only one day I have free, Tuesday. Oh no! Not anymore, I don’t have any free days. It’s exhausting...”* (P1)
Interpersonal Relationships (emergent)	(No quantitative data)	Children value emotional connection with siblings and caregivers in their daily routines.	*“Lately, I enjoy spending more time with my little sister. We have several Disney Channel shows that we watch together because we really like them, and the main characters are sisters. Sometimes we invite my grandma to play with us.”* (P3)
		Shared screen time serves as a means of bonding with family members.	*“When I finish all my lunch, I get to watch TV with my mom... I don’t really like her shows, but at least I get to spend time with her.”* (P15)
		School settings promote peer relationships and social confidence.	*“When I’m at home, I prefer to lock myself in my room. I’m much more interactive and social when I’m at school; I feel freer and more heard.”* (P5)
		Nannies and extended family members often act as primary caregivers in the parents’ absence.	*“When I wake up, the first thing I do is ask my nanny what time it is. She gets my uniform ready, bathes me, combs my hair (...) and takes me to the school bus because mom and dad are working.” (P2)*

Abbreviations: AAP: American Academy of Paediatrics; ICBF: Instituto Colombiano de Bienestar Familiar.

### 3.2. Sleep

**Figure 2.**
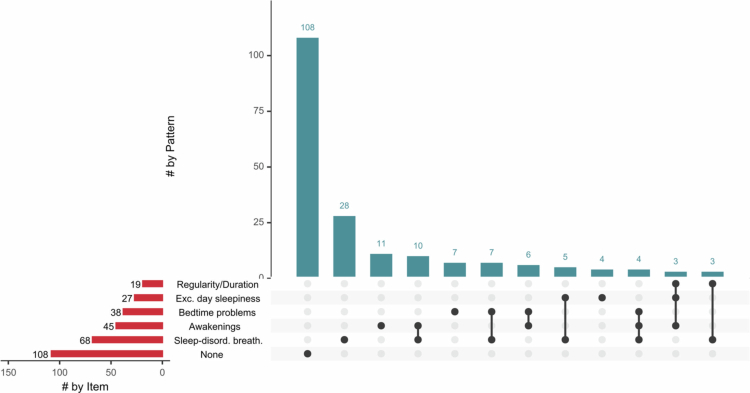
UpSet plot for BEARS sleep disorders domain alterations. Legend: Horizontal bars show the total number of subjects with an alteration in a domain. Vertical bars show the number of sobjects with the pattern of alterations shown below with connected dots. Patterns with fewer than 3 subjects not shown.

Half of the participants (49.7%, *n* = 108) screened positive for at least one domain of sleep disturbance using the BEARS tool. The most frequent issue was sleep-disordered breathing (31.3%), followed by awakenings during the night (20.7%) and bedtime problems (17.5%) (Figure 2). No statistically significant differences were found between males and females.

Children described differing weekday and weekend sleep habits. During school days, bedtimes were usually set by parents, while weekends allowed later sleep, sometimes up to midnight. Several children reported trouble falling asleep and used self-soothing strategies or distractions like reading, eating, or watching TV in bed. These behaviours reflect inconsistent sleep hygiene and how children manage restlessness on their own. Despite these habits, some said they felt rested upon waking, suggesting a gap between perceived rest and the presence of potentially disruptive behaviours (Table II). These accounts indicate that children often normalise sleep issues without seeing them as problems.

Children’s testimonies also revealed the link between daytime overstimulation and nighttime sleep difficulty. A few mentioned falling asleep during the day from fatigue. These narratives highlight how packed schedules—often loaded with academic and extracurricular demands—can indirectly contribute to sleep challenges.

### 3.3. Screen time

All children exceeded the recommended maximum of two daily screen hours. The median total screen time was 6 hours/day (IQR = 4–7). Although no significant differences in overall screen exposure were found between males and females, females reported significantly more entertainment-related screen use (Median = 3 hours; IQR = 2–4) than males (Median = 2 hours; IQR = 1–3; Mann-Whitney U *p* < 0.001). Screen time for educational purposes showed no significant sex differences, with a median of about 3 hours/day.

Children described screen time as a structured part of daily routines, used extensively for both recreation and academics. Some mentioned watching educational content like music tutorials during the week—blurring lines between learning and leisure—and using weekends for movies or series. Screens were also tools for behavioural management; many children noted TV was allowed only after homework or chores. Thus, screen use became a reward system mediated by adults, reinforcing its role as motivator and pacifier. Some children reported watching up to eight hours of TV in one day, yet few expressed discomfort or concern. The duration and centrality of screen use were largely perceived as normal, even expected.

These findings underscore a disconnect between public health guidelines and the realities of children’s routines. Testimonies show that screen time is not just about exposure but also how digital engagement fills structural and emotional gaps in daily life. Addressing excessive use may require broader strategies that consider time availability, parental support, and access to alternative recreational and social opportunities (Table II).

### 3.4. Dietary habits

A large proportion of children reported inadequate fruit and vegetable intake, with 67.7% (*n* = 147) consuming fewer than two servings of fruit per day and 90.3% (*n* = 196) consuming fewer than two servings of vegetables. Males were significantly more likely than females to report low vegetable intake (Chi-squared *p* = 0.026). Regarding beverages, 21.6% (*n* = 47) consumed two or more sugar-sweetened drinks daily. Additionally, 29.0% (*n* = 63) reported nighttime snacking, potentially linked to irregular eating or disrupted sleep. The median number of meals per day was 5 (IQR = 5–5), aligning with general dietary recommendations, though not reflecting meal quality.

Qualitative narratives shed light on how children understand their diets. Despite low fruit and vegetable intake, many viewed their diets as “balanced,” often equating adequacy with variety or traditional completeness. However, their accounts revealed frequent consumption of processed foods (snacks, pastries, sugary desserts), especially in the afternoons. Sweets and baked goods were often framed as routine or rewards, highlighting emotional drivers behind food choices (Table II).

Water consumption, while generally adequate in the quantitative data, was met with ambivalence in interviews. Several children admitted disliking water and preferred flavoured drinks like juice or soda. This suggests a disconnect between reported consumption and actual hydration behaviours, especially when taste preferences outweigh health considerations. Similarly, nighttime snacking (reported by nearly a third) was often described as a coping response to boredom or sleeplessness.

### 3.5. Time management

Though not formally assessed in questionnaires, interviews revealed that most children had highly structured daily schedules. School, homework, extracurriculars, and household routines filled their weekdays. Only limited time was available for unstructured play or rest, mostly reserved for weekends.

Children's accounts reflected not only a lack of discretionary time but also a cumulative sense of physical and emotional fatigue. Several participants reported feeling exhausted by day’s end, with some describing dozing off in class or struggling to stay awake at home. These statements suggest that the intensity and density of daily activities may exceed children's developmental capacity. Moreover, this fatigue seemed internalised as normative; children rarely questioned their schedules, accepting them as inevitable or necessary.

Activities like skating or leisure reading were reported as being “given up” to make room for academic responsibilities. This reflects a broader pattern of time compression, where rest and play become conditional on performance rather than essential parts of daily life. Such compression appears to shape children’s self-perception and identity. Their narratives often reflected a performance-oriented outlook, where productivity was closely tied to feelings of approval, competence, and worth. This dynamic may undermine intrinsic motivation for rest or play, contributing to stress or burnout, particularly if these patterns persist without compensatory moments (Table II).

### 3.6. Interpersonal relationships

Children emphasised the emotional value of time with family, especially siblings, grandparents, or caregivers. Shared routines—watching TV, playing, or eating—were described as moments of closeness. In many households where both parents worked, nannies or grandparents were primary caregivers (Table II).

At home, siblings, grandparents, and nannies played key emotional and practical roles. The situational map illustrates how caregiving responsibilities were distributed among multiple adults, especially when parents’ work limited direct involvement. Grandparents and nannies were often seen not just as caregivers but as co-participants in daily rituals—helping with school prep, meals, or emotional support. Though often overlooked in child well-being assessments, these figures provided stability and affection within shifting post-pandemic structures.

Screen-based bonding was common. Some children described watching TV with a parent as a reward; others highlighted watching shared shows with siblings. Though often seen as isolating, television was portrayed as shared, emotionally meaningful time—marked by mutual enjoyment and symbolic connection. This reframes screen time as a social tool for emotional closeness within busy households.

Peer interactions centred around school, which was seen as a space not just for academics but for freedom and confidence. Several children said they felt “freer,” “more confident,” or “more heard” at school than at home, positioning school as a relational sanctuary where they exercised agency and emotional expression.

**Figure 3.**
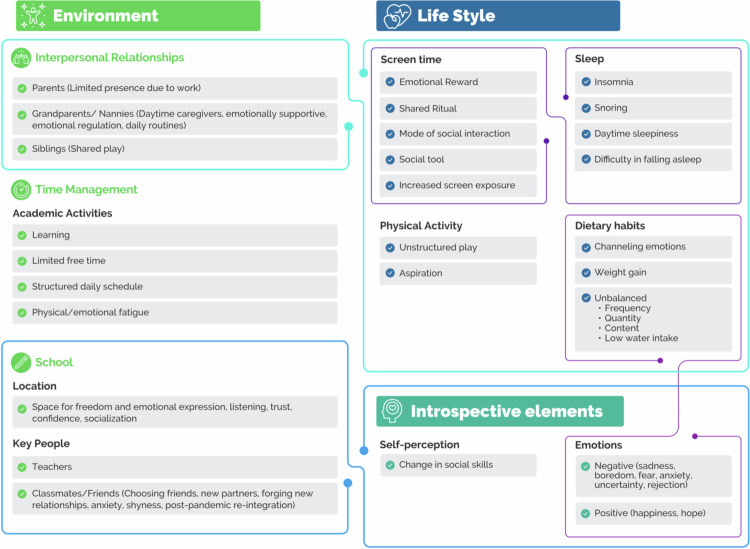
Mapping the Child’s Daily Contexts post-COVID-19. Legend: The fluid movement between care settings (home, school, caregivers) and how time management and interpersonal relationships emerge. Additionally, it interconnects these environments with the child's lifestyle and their introspective elements. It can be observed that the use of screens is a behaviour that aids in falling asleep, which may lead to potential alterations in this area. It is also observed that children report having certain dietary habits based on their emotions.

Not all social experiences were smooth. Some children reported withdrawing in unfamiliar social settings or around peers outside their usual school group. This social hesitation reflects an ongoing recalibration of confidence, possibly shaped by early pandemic-related isolation. Figure 3 illustrates how children’s social worlds span both relational and institutional settings.

## 4. Discussion

The present study offers a comprehensive analysis of children's lifestyle patterns following the relaxation of COVID-19 containment measures in Bogotá, Colombia. The findings reveal not only a partial recovery of pre-pandemic behaviours but also the entrenchment of new post-pandemic norms, many of which diverge sharply from health recommendations.

Physical activity, a core pillar of child health, showed moderate levels among the study population, with approximately two-thirds of children meeting the minimum threshold of engaging in exercise >2 times per week. This is higher than the activity levels recorded during lockdown periods in India (Rajesh et al., 2021) and Spain (Villodres et al., 2023) but remains below pre-pandemic norms. The PAQ-C scores indicated statistically significant sex differences, with boys reporting higher variability and a higher upper-bound for total scores. These findings parallel those of Bartha et al., who reported that Hungarian boys maintained higher post-lockdown activity levels than girls, likely due to greater access to organised sports and fewer domestic constraints (Bartha et al., 2022). Children often framed physical activity as a function of availability, caregiver support, and scheduling conflicts. Many cited school recess and spontaneous sibling play as primary sources of movement, aligning with findings from Polish and Indian studies that noted a replacement of structured sports with unstructured play during the pandemic—a shift that, while beneficial in part, does not fully compensate for the loss of organised exercise (Łuszczki et al., 2021; Narang et al., 2024; Shenoy et al., 2021). Furthermore, several children expressed emotional frustration at their inability to participate in competitive sports, suggesting a gap between aspiration and opportunity that may be particularly pronounced in low-resource settings.

Our findings regarding physical activity align with recent Colombian national data showing widespread challenges in meeting international guidelines among urban children. González et al. (2021) reported that only 35.5% of Colombian children and adolescents meet WHO physical activity recommendations, with particularly low compliance in Bogotá (35% of school-aged children), despite the capital having greater access to structured programs compared to other regions (González et al., 2021). This pattern is consistent with our finding that approximately two-thirds of children met the minimum threshold of exercise ≥3 times per week, yet qualitative data revealed significant barriers related to time constraints, homework demands, and limited opportunities for competitive sports. The Colombian National Survey of Nutrition (ENSIN 2015) documented that urban children face distinct challenges: while 73% of Bogotá children meet sleep duration recommendations, only 35% meet physical activity guidelines, and 77% exceed the recommended screen time limit of 2 hours per day (Ministerio de Salud y Protección Social et al., 2015). These findings underscore that urban Colombian children, like those in our sample, struggle to meet multiple components of the 24-hour movement behaviour guidelines simultaneously. Moreover, consistent with our qualitative findings showing sex-based differences in PAQ-C scores, González et al. demonstrated that girls consistently show lower compliance with activity recommendations across all age groups, highlighting the need for gender-sensitive interventions in Colombian urban settings.

Sleep behaviours in the sample exhibited significant disruption, with nearly half of participants screening positive for at least one domain of sleep disturbance. Sleep-disordered breathing and nighttime awakenings were the most prevalent issues. This is similar to prior observations from Spain and Poland, where lockdown was associated with delayed bedtimes, increased night awakenings, and reduced sleep quality (López-Bueno et al., 2020; Łuszczki et al., 2021). However, the post-lockdown persistence of these problems in the current sample points to a more durable alteration in habits. Children’s narratives reveal efforts to self-manage sleep difficulties through behaviours such as bedtime screen use, late-night eating, or reading, practices known to impair sleep onset latency and overall sleep architecture (Crispim et al., 2011; Grønli et al., 2016).

Interestingly, many children described their sleep as “sufficient” or “normal,” even when problematic. The disconnect between subjective perception and clinical indicators of sleep disturbance suggests that children may have adapted to suboptimal sleep environments, potentially normalising fatigue and poor rest. A similar phenomenon has been described by Łuszczki et al., in which hours of sleep became shorter during the pandemic, despite improvements in the subjective quality of sleep among Polish children (Łuszczki et al., 2021). Although the sleeping habits described in the BEARS screening tool could not be associated with objectively altered sleep architecture or efficiency in our study (i.e. polysomnograms not carried out), their persistence into adulthood is well known to be associated with poor sleep quality, and alterations in physical and cognitive performance (Silvani et al., 2022). The early identification of these maladaptive sleeping habits should prompt primary prevention campaigns to improve sleep hygiene before these behaviours become entrenched.

Screen time was revealed to be one of the behaviours that changed the most, as all children in the study exceeded the maximum recommended by the American Academy of Paediatrics of two hours per day (Hill et al., 2016; Reid Chassiakos et al., 2016). The median daily screen time was six hours, and females reported significantly higher recreational use than males. This is consistent with post-pandemic data from several countries, including Spain and Brazil, where screen time remained persistently high due to ingrained digital habits formed during lockdown (Amaral de Andrade Leão et al., 2025; Villodres et al., 2023). In particular, the dual function of screens, as educational tools and entertainment media, complicates the interpretation of exposure. Several children in the present study described screen time as a regulated reward, access to which depended on completing homework or good behaviour. This instrumentalization of screen use, while effective for behaviour control, risks reinforcing excessive dependence and framing digital consumption as compensatory behaviour. Qualitative findings from pior work indicate that when screens are used as rewards or to manage routines, they become emotional regulators, displacing key developmental experiences such as play and social interaction (Azevedo et al., 2025). This may undermine self-regulation and contribute to reduced school readiness and attention skills.

The data reveal a qualitative shift in the role of screens from solitary activity to social practice. Children frequently described screen use as shared—watching TV with siblings or parents, using media to bond or decompress. This nuance complicates the traditionally negative framing of screen time. Indeed, a recent systematic review found that co-viewing is positively associated with cognitive outcomes such as executive function, language, and academic skills, and may also be linked to improved psychosocial indicators, including fewer internalising and externalising behaviours and greater socio-emotional competence (Mallawaarachchi et al., 2024). Nevertheless, the normalisation of excessive screen use—particularly when not balanced by physical or interactive activities—remains a concern (Guellai et al., 2022).

Dietary behaviours in the study population reflected a striking dissonance between perception and practice. While most children viewed their diets as “balanced,” quantitative indicators revealed widespread deficiencies. Over 90% consumed fewer than two servings of vegetables daily, and more than two-thirds failed to meet fruit intake recommendations. These patterns are comparable to those found in Polish, Japanese, Australian and Spanish cohorts, where snack consumption and sugar-sweetened beverage intake rose significantly during the pandemic and have yet to decline (López-Bueno et al., 2020; Łuszczki et al., 2021; Smout et al., 2024; Sugimoto et al., 2023). Notably, 29% of our sample reported nighttime snacking, a behaviour often associated with emotional regulation, disrupted sleep, or irregular meal timing (Lepley et al., 2022; Zhao et al., 2023). Qualitative data supported this interpretation, with children citing boredom, stress, or difficulty sleeping as motivations for late-night eating.

The persistence of sugar-sweetened beverage consumption observed in our study aligns with findings from Sylvetsky et al., who reported that children’s increased intake of these beverages during the COVID-19 pandemic was driven by emotional needs, greater availability at home, and disrupted routines (Sylvetsky et al., 2022). Our post-pandemic data suggest that these habits were not transient: several children still reported a dislike for water and a clear preference for juices or flavoured drinks—a pattern also noted in international samples (Bartha et al., 2022; López-Bueno et al., 2020; Łuszczki et al., 2021; Sugimoto et al., 2023). Although many children articulated an understanding of healthy nutrition, their narratives revealed frequent consumption of processed foods, undervaluation of the negative constituents of desserts, and strong emotional associations with certain foods, such as sweets as rewards and snacks as comfort. These findings emphasise the need for nutritional interventions that address not only knowledge gaps but also emotional regulation, routine rebuilding, and family food practices (Franse et al., 2019).

Although not part of the quantitative design, the category of time management emerged strongly in interviews. Children consistently described highly structured routines dominated by school, homework, and chores, with limited space for unstructured play or rest. Reports of fatigue, falling asleep during class, or abandoning leisure activities to prioritise academic work—or even extracurricular demands—indicate a broader pattern of time compression. This is in line with literature on academic childhood stress, which highlights how the drive to “make up” for perceived academic loss has intensified schedules, often at the expense of psychosocial recovery (Iuga et al., 2023; Morando et al., 2023).

Importantly, interviews revealed a trend among families from middle- and high-income backgrounds to enrol their children in multiple extracurricular programs, often as a compensatory response to time “lost” during the pandemic and as a means of providing opportunities that parents themselves lacked in their own childhoods (Brown et al., 2011; Velija & Allen, 2024). The increasing accessibility of mobile internet and on-demand educational platforms has further enabled this phenomenon, allowing impulsive enrolment in structured activities without institutional mediation (Zhang et al., 2024). As a result, children’s time is saturated with productivity-oriented tasks, frequently leaving little room for free play, spontaneous rest, or emotional decompression. These patterns intersect with recent findings on post-pandemic academic burnout, which indicate that children and adolescents—particularly those with high self-expectations or exposed to external performance pressures—may internalise ideals of constant achievement, leading to emotional exhaustion and reduced well-being (Morando et al., 2023). The internalisation of performance norms was evident in children’s language: many linked their value to productivity, framing play as a luxury contingent on task completion. These findings raise serious concerns about the psychological toll of overscheduled childhoods and suggest that public health messaging should explicitly reframe rest and play as developmental necessities rather than optional rewards.

Finally, the structure and quality of interpersonal relationships underwent notable transformation. Children reported a reliance on distributed caregiving networks, including grandparents and nannies, as parents returned to full-time work. These figures provided not only practical support but also emotional continuity, especially during times of uncertainty, mirroring evidence that multigenerational support sustained family well-being during the pandemic (Gilligan et al., 2020; Sudo et al., 2025). Despite this continuity, children paradoxically noted that they missed spending undistracted quality time with parents when they were physically present. School environments, on the other hand, were frequently described as emotionally safer than home: children felt “more listened to” and “freer” in the classroom. This aligns with literature positioning schools as vital arenas for post-pandemic emotional recovery and community rebuilding, not merely for academic instruction (e.g., school re-entry programs and fostering school belonging) (Roffey, 2023).

### 4.1. Limitations and future research

Several limitations in our study must be considered. First, the cross-sectional design restricts the ability to draw causal inferences and limits insight into temporal or developmental dynamics. Second, the use of self-reported data—particularly from children—may be influenced by recall bias or social desirability, even though qualitative methods aimed to encourage authenticity. Third, the urban school-based sample almost certainly does not capture the experiences of children in rural settings or those with limited access to formal education.

Despite these limitations, the study identifies multiple areas where public health, education, and policy can intervene to restore balance in children’s daily lives. Interventions should not aim solely to recover pre-pandemic habits, but to reconfigure daily environments in ways that promote long-term resilience. Schools, as emotionally significant spaces for many children, must be equipped not only for academic instruction but also for social reintegration. Prioritising access to structured physical activity, monitoring sedentary time, and safeguarding rest through evening screen curfews are concrete targets for school-based programs. Nutrition initiatives should integrate emotional regulation strategies, acknowledging that food behaviours are often shaped by affective needs rather than informational deficits. Most importantly, rest and play should be publicly reframed, not as optional leisure, but as essential developmental resources.

## 5. Conclusion

This mixed-methods study reveals that urban Colombian children's post-pandemic lifestyle patterns reflect normalised health-risk behaviours rather than simple lapses in guideline adherence. While two-thirds met minimum physical activity thresholds, qualitative narratives uncovered significant barriers including time compression, homework demands, and socioeconomic limitations preventing engagement in desired activities. Nearly half exhibited sleep disturbances yet perceived their rest as adequate, a concerning normalisation given long-term consequences on development. Universal non-compliance with screen time recommendations (median 6 hours daily) was not experienced as problematic, with screens described as integrated tools for learning and family bonding. Despite widespread nutritional inadequacies (over 90% consuming insufficient vegetables), children perceived their diets as balanced, highlighting a critical disconnect between health literacy and practice.

Two emergent themes (time management and interpersonal relationships) provide essential context missing from surveillance data. Children described productivity-oriented schedules leaving minimal space for unstructured play, with fatigue internalised as normative. Reliance on distributed caregiving networks and the emotional significance of school environments underscore that post-pandemic recovery extends beyond behavioural correction to encompass relational reconfiguration.

These findings call for integrated strategies addressing emotional regulation, revaluing rest and play as developmental necessities, and reconfiguring environments to support child-centred routines. Future research should employ longitudinal designs, incorporate objective 24-hour movement measures, and expand to rural populations while addressing the structural and affective dimensions inseparable from children's daily lives.

## Supplementary Material

Supplementary Material Supp1.docx

## Data Availability

The original contributions presented in the study are included in the article, further inquiries can be directed at the corresponding author. The raw data supporting the conclusions of this article will be made available by the corresponding author only if this request is approved by the Research Ethics Committee of Fundación Universitaria Sanitas, Bogotá D.C., Colombia, following current legislation regarding patient protection in Colombia.
