# Supplementary material for: Physical activity, sleep, dietary habits, and screen time in Bogotá’s children post-pandemic lockdown: a mixed-methods study
Source: Int J Qual Stud Health Well-being. 2026 Jun 13;21(1):2683936. doi: 10.1080/17482631.2026.2683936 (PMC13267027; doi:10.1080/17482631.2026.2683936)
Supplement: Supplementary Material — Supp1.docx [file ZQHW_A_2683936_SM8841.docx]

**Supplemental 1.** Semi-structured interview Guide

1. Let’s start by talking about your daily routine. Can you describe a typical day for you, from the moment you wake up until you go to bed? Is your routine different on weekdays compared to weekends?
2. Now think about a recent experience that was special for you. Can you tell me about a moment in the last month that you really enjoyed, either with your family, with friends, or at school?
3. What kinds of activities do you do to get your body moving or to play? Where do you usually do them and who do you do them with?
4. Let’s talk about screens and devices. What do you usually watch or do using screens (TV, phone, tablet, computer)? How much time do you think you spend on screens during a normal day? Does the amount of time change between weekdays and weekends?
5. Now let’s talk about food and meals. What are your meals like throughout the day? What do you usually eat and at what times? Is there anything you eat more often or something you never miss during the day? Do you have fixed times for meals, or does it change depending on the day?
6. What time do you usually go to sleep on school days? Do you do anything before going to bed, like reading, watching something, or eating? Is your bedtime or night routine different on weekends?
7. Do you feel like you have enough time to rest, play, or relax during the day? Are there things that make it hard for you to have free time?
8. Is there anything else you’d like to tell me about your daily life, your routine, or something you think is important and we haven’t talked about?
